# Significance of Methylation of FBP1 Gene in Non-Small Cell Lung Cancer

**DOI:** 10.1155/2018/3726091

**Published:** 2018-06-10

**Authors:** Yao Dong, Sheng Huaying, Wan Danying, Zhu Chihong, Jiang Ruibin, Sun Xiaojiang, Feng Jianguo

**Affiliations:** ^1^Cancer Research Institute, Zhejiang Cancer Hospital, Hangzhou, Zhejiang 310022, China; ^2^Key Laboratory Diagnosis and Treatment Technology on Thoracic Oncology, Hangzhou, Zhejiang 310022, China

## Abstract

Because NSCLC has poor overall prognosis and is frequently diagnosed at later stage, we aimed to seek novel diagnosis biomarkers or therapy target of the disease in this study. Fructose-1,6-bisphosphatase 1 (FBP1) is a rate-limiting enzyme in gluconeogenesis, which was usually lost in NSCLC due to abnormal methylation in promoter DNA sequence. The clinical data indicated that the methylation rate in FBP1 gene promoter was negatively related to the overall survival of the NSCLC patients. DNA methylation transferase inhibitor 5-aza treatment could significantly increase both expression levels of mRNA and protein in A549 cell line. On the other hand, silence of FBP1 in H460 cell line by using specific siRNA against FBP1 dramatically improved the cell proliferation and cell migration according to the date of FACS and transwell assays. All these findings implied the important roles of FBP1 expression in lung cancer development and progression and the potential use of the methylation status detected in FBP1 promoter region as a novel predictor for prognosis and therapeutic target for NSCLC patients.

## 1. Introduction

Lung cancer is the leading cause of cancer-related death worldwide. In 2017, it is estimated that 155,870 people will die of this disease in the United States [1]. Non-small cell lung cancer (NSCLC) is the most common type and accounts for up to 85% of all lung cancer cases [2–4]. The overall prognosis of lung cancer is poor due to late-stage detection and ineffective therapies, and the 5-year overall survival rate of NSCLC is only 15% across all stages [5, 6].

Emerging evidences have shown that genetic alteration and aberrant expression become increasingly important as diagnosis markers and predictors of treatment or for genetic aberration-based personalized medication or development of new treatment strategies that could benefit NSCLC patients [7, 8]. Epigenetic alterations through DNA methylation or histone modifications that influence the gene expression without changing DNA sequence have been demonstrated to be as important as genetic mutations in NSCLC. For example, frequent DNA hypermethylation resulting in gene silence of numerous critical tumor suppressor genes, such as p16, MGMT, DNPK, and APC, has been found to be associated with development and progression of NSCLC [1, 9].

Studies have also been focusing on the abnormal glycolysis in cancer cells after characterization of the “Warburg Effect”. Fructose-1,6-bisphosphatase 1 (FBP1) is a rate-limiting regulatory enzyme in gluconeogenesis that can catalyze the hydrolysis of fructose 1,6-bisphosphate to fructose 6-phosphate in the presence of divalent cations. Loss of FBP1 results in glycolytic flux and glucose uptake and maintenance of ATP production under hypoxia [10–12], leading to hypoglycemia and lactic acidosis in patients. Loss of FBP1 expression in cancer cells has shown a critical role as oncogenic driver in EMT and BLBC [10, 13]. Lower FBP1 expression has been detected in multiple cancers, including hepatocellular, colon, gastric cancer, basal-like breast cancer (BLBC), clear cell renal cell carcinoma (ccRCC), and pancreatic cancer [10, 13–19], and correlated with advanced tumor stages and worse patient prognosis. Studies have also revealed that reduced FBP1 expression in hepatocellular, colon, and gastric cancer is caused by DNA hypermethylation in its promoter region [15, 16]. In breast cancer patients, DNA methylation in the promoter region of FBP1 also decreased FBP1 expression in liver tissue [20]. In addition, our previous study showed that reduced FBP1 levels at both mRNA and protein are a negative prognostic molecular maker for NSCLC. These results indicated the importance of FBP1 involved in cancer development and progression; however, the detailed mechanism is still unclear [21–23]. In the current study, we showed that the promoter DNA of FBP1 is hypermethylated in NSCLC tissues compared with normal tissues. Furthermore, high level of methylation in FBP1 promoter is negatively correlated with overall survival rates of NSCLC patients. Of interest, we also found that FBP1 silence increases the percentage of cell in S-phase and promotes the cell migration.

## 2. Materials and Methods

### 2.1. Patients and Tissue Collection

We enrolled a cohort of 107 (male=90, female=17) patients with histopathologically confirmed NSCLC diagnosis at Zhejiang Cancer Hospital between March 2008 and April 2010. Fresh tumor and paired control samples were obtained during operation and rapidly frozen in liquid nitrogen. The paired adjacent normal tissues were obtained at least 2 cm from the tumors. All the tumor samples comprised at least 70% tumor tissues for molecular studies confirmed with HE staining. Key exclusion criteria for this study included a history of pneumonitis, chemotherapy and/or radiotherapy, or other cancer treatments before operation. At the time of surgery, patients' age ranged from 37 to 75 years, with average of 59.6±8.3 years. The last follow-up data was collected in November 2015. The histological classification was based on the WHO/IASLC classification criteria for lung tumors, squamous cell carcinoma: 70 cases and adenocarcinoma: 37 cases. The stage was classified according to the guidelines of the 7th edition of TNM staging in lung cancer, stage I: 43 cases (Ia=6, Ib=37), stage II: cases (IIa=1, IIb=31), and stage III: 32 cases (IIIa=29, IIIb=3).

Written informed consent from the subject patients was obtained for the use of these samples in research and the protocol approval was obtained from the Clinical Research Ethics Committee of the Zhejiang Cancer Hospital.

#### 2.1.1. DNA Preparation and Methylation Analysis

Genomic DNA was isolated from 2mm3 homogenized samples using DNeasy Blood and Tissue Kit (Qiagen, Valencia, CA, USA) according to the manufacturer's instructions. Genomic DNA is bisulfite converted, and then the FBP1 promoter region is amplified via PCR. Methylation status of the FBP1 promoter region was evaluated by pyrosequencing analysis [24] performed on PyroMark Q96 ID (Qiagen, Valencia, CA, USA) in this study. PCR primer sequences, PCR conditions, and sequencing primer sequences are available upon request. The mean methylation across all CpG sites was analyzed and calculated for each sample and represented as the methylation rate.

#### 2.1.2. Cell Culture

A549 and H460 cells were obtained from ATCC. A549 cells are grown in RPMI-1640 and H460 cells in F12 and both are supplemented with 10% FBS (Life Tech, #16000044) and 1x penicillin/streptomycin mix. Cells were grown in a cell culture incubator at 37°C, 5% CO2, 100% relative humidity. Transfections were conducted with using LipofectamineTM 2000 transfection reagent according to the manufacturer's protocol.

### 2.2. mRNA and Protein Isolation

Cells were grown to 50-80% confluence after being seeded about 24 hours, at which time transfection was performed or 5-Aza were added. After another 24-hour incubation, cells were harvested and mRNA and protein samples were harvested with AllPrep DNA/RNA/Protein Mini Kit (QIAGEN, #80004) following the manufacturer's protocol. The obtained mRNA was quantified on NanoDrop 2000 and further used to generate cDNA. qPCR was performed with 2ng of cDNA by using SYBR Green method according to manufacturer's instruction (T*aKaRa Ex Taq*® HS, 420A, Japan), combined with GADPH as the internal control. The primer sequences for FBP-1 (forward: 5′-AGGAAGCACAAAGCCAAGTGAAGG-3′; reverse: 5′-TGAGGATGAAGTGACCTTGGGCAT-3′) and GAPDH (forward: 5′-TGAAGGTCGGAG TCAACGGATTTGGT-3′; reverse: 5′-CATGTGGGCCATGAGGTCCACCAC-3′).

### 2.3. Western Blotting

Total protein was quantitated by BCA assay (Thermo Scientific, #23225). Samples were boiled for 5 min with 1XSDS sample loading buffer prior to loading. Samples were run on 10% SDS-PAGE gels after which proteins were transferred to polyvinylidene difluoride membranes; then they were blocked in 5% (w/v) fat-free milk-PBST (phosphate buffer with 0.05% Tween 20) for 1 hour at room temperature. The membranes were incubated with rabbit anti-FBP1 monoclonal antibody (Abcam, # ab109732) or anti-GAPDH antibody (Abcam, #ab8245) in PBST with 5% BSA overnight at 4°C. The membrane was washed three times with PBST and then incubated in HRP-anti-mouse IgG or HRP-anti-rabbit IgG secondary antibodies diluted 1  : 5000 in PBST with 5% (w/v) fat-free milk for 1 hour at room temperature. After washing three times with PBST, the specific bands were developed on the films via using SuperSignal West Pico Chemiluminescent Substrate (Thermo Scientific).

### 2.4. FACS Assay

Cells were collected and washed with cold PBS twice and fixed with cold 70% ethanol overnight. Cells were then centrifuged and washed with cold PBS twice again. Resuspended cell pellet in PBS and stained cells with 50 *μ*g/ml PI. After incubation for 30 min at 4°C, cells were analyzed with flow cytometry (Beckman Coulter CytomicsTM FC 500, Brea, California).

### 2.5. Transwell Assay for Cell Invasion

The cell invasion assays were performed using transwell chambers with 8-*μ*m pores (Corning Incorporated) coated with Matrigel matrix (BD Biosciences). Cells were transfected with NC or siRNA-FBP-1. 48 hours later after transfection, cells were collected and resuspended in culture medium without FBS. Transfected cells (5x104) FBS-free medium was added to the upper chambers, and 500 *μ*l culture medium containing 20% FBS was added to the lower chambers. After incubation at 37°C for 16 h, the transwell chambers were stained with 0.5% crystal violet for 10 min. Nonmigrated and noninvaded cells were removed using cotton swabs. Migrated and invaded cells were imaged and counted using an inverted microscope (Olympus Corporation). The experiment was repeated at least three times.

### 2.6. Statistical Analysis

All statistical calculations were conducted with the use of SPSS13.0 statistical software. Two-sided Fisher's exact tests were used to evaluate associations between tumor mutations and age, Dukes' staging, gender, and tumor location.

## 3. Results

### 3.1. FBP1 Promoter Methylation Correlates with Different Clinical-Pathological Factors including Histologic Grade in the Cancer and Normal Tissues of the NSCLC Patients

**Figure 1:**
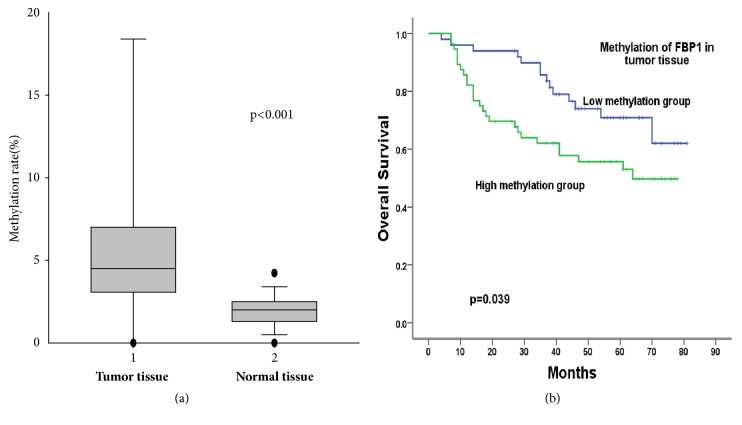
Methylation status of FBP1 promoter detected in lung cancer and normal tissues and its relationship with overall survival. (a) FBP1 methylation was assessed in 107 pair lung cancer and normal tissues using methylation assay. (b) Statistical analysis was performed to confirm the correlation between FBP1 methylation and overall survival rate of lung cancer patients.

We previously showed that DNA methylation in the promoter region might contribute to the lower mRNA level [21]. In this study, we utilized the pyrosequencing analysis to further validate the mechanism of lower level mRNA of FBP1 in cancer tissues. Of note, we detected methylation in FBP1 promoter in all tested human specimen. We thus used the medium methylation rate detected in tumor tissues as a cut-off value for FBP1 promoter region methylation level**. To this setting, **DNA methylation found in FBP1 promoter region of cancer samples (7.31% ± 0.095) was significantly higher than control (2.31% ± 0.021, p<0.001, Figure 1(a)). Of note, the DNA methylation level of FBP1 promoter region corresponds to the mRNA level change [21].

We also determined the possible correlations of DNA methylation of FBP1 promoter with NSCLC patient`s overall survival and disease progression (Table 1). Our results showed that, with using the cut-off value set-up for FBP1 promoter region methylation level, FBP1 promoter methylation was negatively associated with overall survival. The methylation ratio is also reversely correlated with the tumor differentiation, and well-differentiated tumors (high to middle grade) have statistically significant lower level of FBP1 methylation comparing to middle to low grade of tumors (p<0.01). Smoking is the biggest cause of lung cancer, and our results showed the methylation level in the cancer tissue of smokers is significantly higher than nonsmokers' (p<0.001). Results of nonparametric and Chi-Square test also indicated the association between FBP1 promoter methylation and sex, whereas the methylation ratio is higher in male than female. However, the methylation status of the FBP1 promoter region has no correlations found with other factors including age, family history, drinking, histologic type, and clinical stage.

Next, we examined the FBP1 DNA methylation level in the normal adjacent tissues of the NSCLC patients and analyzed the relationship of methylation rate with clinical-pathological factors. As shown in Table 2, both nonparametric and Chi-Square tests showed a positive relationship (p<0.05) between FBP1 DNA methylation and smoking history. Together with the results revealed from cancerous tissues of smoking patients, our date indicate that smoking may cause increase of methylation in FBP1 promoter region. Of interest, nonparametric test also indicated a strong correlation of FBP1 DNA methylation increase and family history (p=0.016); however, no such association was found in these enrolled patients with chi-Square test. Other clinical-pathological factors, like age, sex, histologic type, grade, clinical stage, and drinking, did not show the relationship with FBP1 DNA methylation.

### 3.2. DNA Methyltransferase Inhibitor 5-Aza-CdR Effectively Rescues the FBP1 Gene Expression in NSCLC Cells

**Figure 2:**
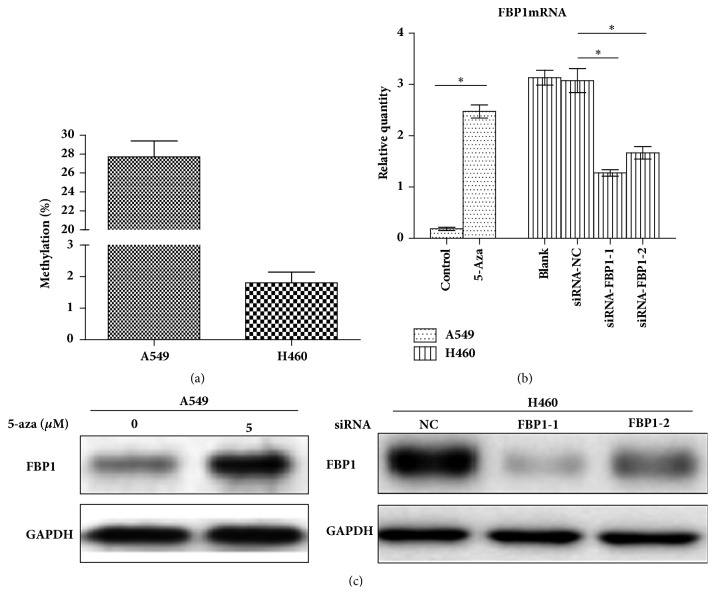
Effects of 5-Aza treatment on FBP1 expression in lung cancer cells. (a) Graph showing the the mRNA levels determined in A549 and H460 cells; (b) graph showing changes of FBP1 protein levels in A549 and H460 cells treated with 5-Aza or SiRNA-FBP; (c) Western blot results showing the changes of FBP1 protein expression in cells with indicated treatment. The data represents the average of the results from three independent experiments. Error bar indicates the standard deviation. “*∗*” indicates P<0.05.

We next determined whether the silence of FBP1 expression in NSCLC cells is mainly caused by DNA hypermethylation in promoter. For this, we used established human lung cancer cell line A549 and H460 cells. We first examined the methylation level of FBP1 promoter region of these two cell lines and found that the mean methylation rate is 28.3% for A549 cells and 1.8% for H460 cells, respectively (Figure 2(a)). As expected, we also observed that A549 cells had low FBP1 expression, while H460 cells showed relative higher expression of FBP1 protein. We then exposed A549 cells to 5-Aza-CdR to demethylate FBP1 promoter DNA methylation and found that 5-Aza-CdR treatment upregulates FBP1 expression in both mRNA and protein levels (Figure 2). These results thus further validated that the DNA methylation in the promoter region reduces expression of FBP1 in human cancer cells.

### 3.3. Methylation of FBP1 Promoter Region Significantly Impacts Antitumor Effects in NSCLC Cells

**Figure 3:**
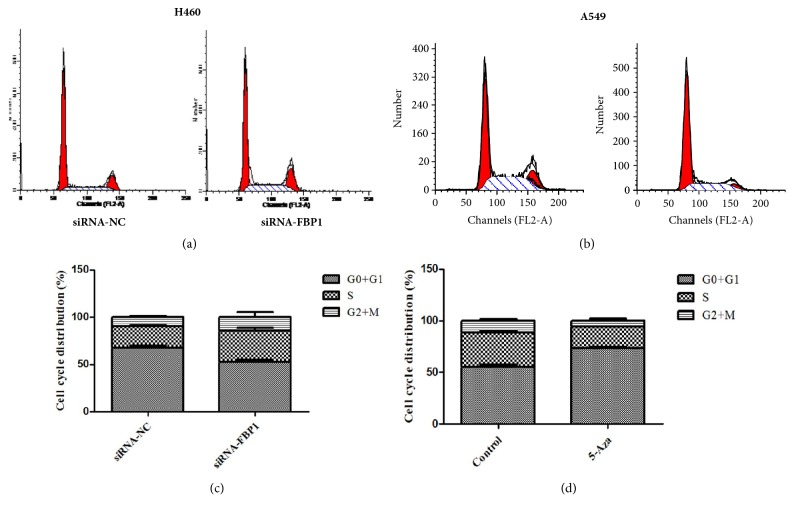
Downregulation of FBP1 induces cell cycle arrest in H460 cells. Cells were transfected with siRNA against FBP1; A549 cells that are with high methylation level are detected in the FBP1 promoter region; we observed treatment with 5-Aza-CdR; and cell cycle distribution was analyzed by FACS assay. (a, c) Representative results of cell cycle analysis; (b, d) graph showing the changes of cell cycling in H460 and A549 cells. The data represents the average of the results from three independent experiments. Error bar indicates the standard deviation.

To further explore the underlying roles of FBP expression and methylation of FBP1 promoter region in NSCLC, we first used specific small interfering RNA (siRNA) to downregulate the endogenous level of FBP1 expression in H460 cells that express higher level of FBP1 (Figure 2) and tested the consequences of knocking down of FBP1 expression on cell cycling. We found that knocking down of FBP1 expression via siRNA in H460 cells significantly increased the accumulation of S-phase cells and decreased the percentage of G0-G1 phase cells of H460 (Figures 3(a) and 3(b)). In A549 cells that are with high methylation level detected in the FBP1 promoter region, we observed that treatment with 5-Aza-CdR caused dramatic increase of the percentage of G0/G1 (Figures 3(c) and 3(d)). These results suggest that methylation status of FBP1 promoter region, or FBP1 expression, affects cell cycling of human lung cancer cells.

**Figure 4:**
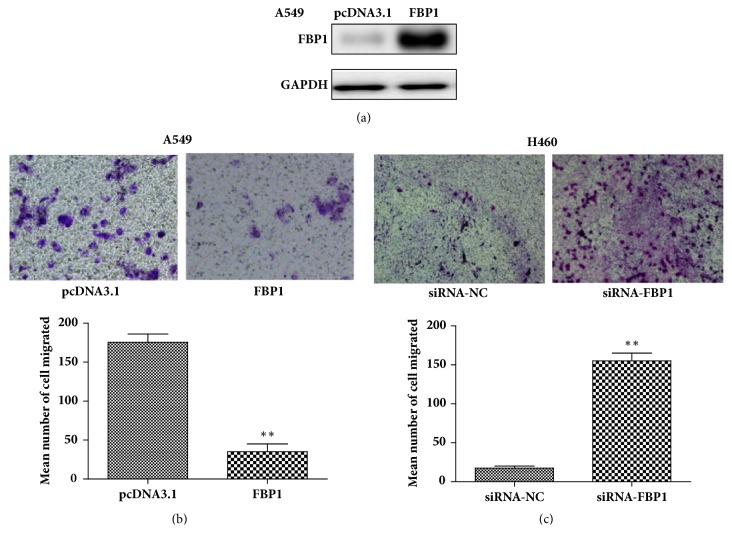
Effect of FBP1 expression on cell invasiveness of A549 and H460 cells. (a) Western blot result shows the overexpression of FBP1 protein in A549 cells; (b and c) representative images showing the engineered FBP1 expression in A549 cells (b) and SiRNA-knockdown of FBP1 expression in H460 cells (c) on cell invasiveness. Graphs showing the changes of cell invasiveness of A549 cells (b) and H460 cells (c). The data represents the average of the results from three independent experiments. Error bar indicates the standard deviation. “*∗*” indicates P<0.01.

**Figure 5:**
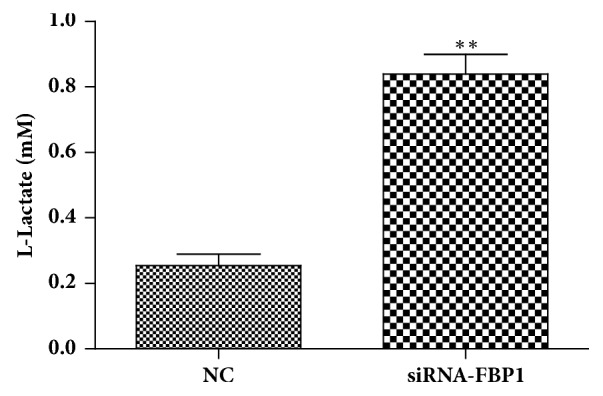
Knock-down of FBP1 expression increases L-lactate level in H460 cells. The data represents the average of the results from three independent experiments. Error bar indicates the standard deviation. “*∗*” indicates P<0.01.

Previous studies have shown that the activity of fructose-1,6-bisphosphate aldolase A promoted the metastasis and migration in the lung squamous cell carcinoma [24–27]. We thus performed transwell assays to test the potential role of FBP1 expression on cancer cell invasiveness. In this experiment, we used A549 and H460 cells with engineered FBP1 expression. Our results showed that overexpression of FBP1 dramatically reduced invasiveness of A549 cells, and siRNA-induced downregulation of FBP1 expression in H460 cells increased ability of invasiveness (Figure 4). These results suggest that FBP1 expression is involved in the NSCLC invasion process. In addition, we further noticed that siRNA treatment increased L-lactate level in H460 cells, indicating regulatory role of FBP in glucose metabolism of cancer cells (Figure 5).

## 4. Discussion

Our previous study revealed that FBP1 mRNA was significantly decreased in lung cancer tissues compared with normal tissues [21]. In addition, the patients with higher level of FBP1 RNA expression have significantly longer disease free survival and overall survival as compared to the lower expression groups [25–27]. In the current study, we determined the underlying mechanism for FBP1 expression suppression and its biological functions in lung cancer cells. Due to high rate CpG islands located in FBP1 gene promoter region and its possible regulation mechanisms as previously revealed [25–27], we investigated the methylation status of FBP1 promoter region in paired human lung cancer and adjacent normal tissues. Our data showed significantly high rate of methylation in FBP1 promoter in lung cancer tissues verses paired normal tissues, and the detected higher methylation level also corresponds to lower FBP1 expression. We also found that treatment with 5-Aza-CdR, a DNMT1 inhibitor, could recover the FBP1 expression in both mRNA and protein levels. These findings suggest that DNA hypermethylation is a dominated factor for FBP1 expression regulation in lung cancer. On the other hand, we found that the lower methylation of FBP1 in cancer tissues is associated with better overall survival for lung cancer patients, indicating a potential of methylation level in FBP1 promoter as a novel predictor for prognosis in non-small cell lung cancer patients. In addition, our results showed that treatment with siRNA against FBP1 downregulated FBP1 expression and consequently promoted the cell proliferation and cell invasiveness and enhanced glycolysis flux in cancer cells, suggesting that FBP1 expression can be considered as an antitumor molecular target in lung cancer.

As mentioned above, the detailed mechanisms of antitumor function of FBP1 are still remaining to be unclear; it has elucidated that Snail-G9a-Dnmt1 complex is critical for FBP1 silence, which promoted the interaction of *β*-catenin and TCF and played an important role in EMT transformation in basal-like breast cancer [25–27]. Recently, Li* et al.* found that FBP1 downregulation could enhance the activity of Wnt/*β*-Catenin pathway and increase the level of its downstream targets, including c-Myc and MMP7 in human breast cancer cells [25–27], suggesting that FBP1 might take part in regulating cancer cell migration via Wnt/*β*-catenin signaling pathway. On the other hand, FBP1 could decrease glycolytic flux in renal tubular epithelial cells and subsequently inhibits the Warburg effect in lung cancer cells as predicted. However, FBP1 restrains cell proliferation, glycolysis, and the pentose phosphate pathway by inhibiting nuclear HIF function via direct interaction with the HIF inhibitory domain in clear cell renal cell carcinoma cells [25–28]. These findings, together with the data present in this study, indicate the important roles of FBP1 expression in cancer development and progression, and the methylation level in FBP1 promoter can serve as a novel biomarker for prognosis and therapeutic target for NSCLC patient. However, future studies are needed to further determine the underlying mechanisms for anticancer activity of FBP1.

## Figures and Tables

**Table 1:** Association of FBP1 DNA methylation level with clinicopathological factors in the cancer tissue of patients with NSCLC.

Factors	Patients, n(%)	FBP1	*P*	FBP1		*P*
		Median (Mean, 5th-95th)		Low methylation n( % )	High methylation n( % )	
Sex			***0.026***			***0.009***
Male	90(84.11)	4.86(7.86,5.76-10.00)		58(54.21)	52(48.60)	
Female	17(15.89)	3.75(4.40,1.76-7.03)		13(12.15)	4(3.74)	
Age(years)			0.341			0.464
<65	77(71.96)	4.50(6.70,5.07-8.32)		35(32.71)	42(39.25)	
≥65	30(28.04)	4.13(8.91,3.74-14.10)		16(14.95)	14(13.09)	
Family History			0.752			0.817
no	87(81.31)	4.50(6.51,4.93-8.09)		41(38.31)	46(42.99)	
yes	20(18.69)	4.38(10.81,3.57-18.06)		10(9.35)	10(9.35)	
Smoking			0.003			***0.008*** ^*∗∗*^
Never	22(20.56)	3.50(3.83,1.97-5.69)		16(14.95)	6(5.61)	
Ever/Current	85(79.44)	5.25(8.22,6.00-10.43)		35(32.71)	50(46.73)	
Alcohol			0.290			0.164
Never	45(42.06)	4.25(6.52,3.71-9.34)		25(23.36)	20(18.69)	
Ever/Current	62(57.94)	5.13(7.89,5.44-10.33)		26(24.31)	36(33.64)	
Histologic type			0.160			0.579
Squamous cell carcinoma	37(34.58)	4.25(5.01,3.09-6.93)		19(17.76)	18(16.82)	
Adenocarcinoma	70(65.42)	5.00(8.53,5.95-11.11)		32(29.91)	38(35.51)	
Grade			***0.073***			***0.004*** ^*∗∗*^
high-middle	58(54.21)	4.00(7.47,4.53-10.42)		35(32.71)	23(21.50)	
middle-low	49(45.79)	5.50(7.12,5.09-9.15)		1614.95()	33(30.84)	
Clinical stage			0.952			0.760
I - II	83(77.57)	4.50(7.23,5.22-9.24)		36(33.64)	38(35.51)	
III	24 (22.43)	4.38(7.60,3.10-12.11)		15(14.02)	18(16.82)	

**Table 2:** Association of FBP1 DNA methylation level with clinicopathological factors in the normal tissue of patients with NSCLC.

Factors	Patients, n(%)	FBP1	*P*	FBP1		*P*
		Median (Mean, 5th-95th)		Low methylation n( % )	High methylation n( % )	
Sex			0.566			0.430
Male	90(84.11)	2.13(2.31,1.89-2.73)		43(40.19)	47(43.93)	
Female	17(15.89)	2.00(2.31,0.98-3.63)		10(9.35)	7(6.54)	
Age(years)			0.277			0.423
<65	77(71.96)	2.00(2.25,1.74-2.77)		40(37.38)	37(34.58)	
≥65	30(28.04)	2.25(2.45,1.84-3.06)		13(12.15)	17(15.89)	
Family History			0.016			0.653
no	87(81.31)	2.00(2.22,1.81-2.62)		44(41.12)	43(40.19)	
yes	20(18.69)	2.40(2.71,1.40-4.03)		9(8.41)	11(10.28)	
Smoking			0.021			*0.014* ^*∗∗*^
Never	22(20.56)	1.58(1.41,0.97-2.22)		15(14.02)	7(6.54)	
Ever/Current	85(79.44)	2.84(3.32,2.81-4.21)		33(30.84)	52(48.60)	
Alcohol			0.196			0.781
Never	45(42.06)	2.00(2.28,1.67-2.89)		23(21.50)	22(20.56)	
Ever/Current	62(57.94)	2.13(2.33,1.78-2.88)		30(28.04)	32(29.91)	
Histologic type			0.320			0.784
Squamous cell carcinoma	37(34.58)	2.25(2.28,1.65-2.91)		19(17.76)	18(16.82)	
Adenocarcinoma	70(65.42)	2.00(2.33,1.96-2.86)		34(31.78)	36(33.64)	
Grade			0.118			0.916
high-middle	58(54.21)	2.00(2.09,1.60-2.58)		29(27.10)	29(27.10)	
middle-low	49(45.79)	2.25(2.57,1.89-3.24)		24(22.43)	25(23.36)	
Clinical stage			0.039			0.181
I - II	83(77.57)	2.00(2.24,1.79-2.70)		44(41.12)	39(36.45)	
III	24 (22.43)	2.25(2.53,1.62-2.53)		9(8.41)	15(14.02)	
